# Hybrid wheat: quantitative genetic parameters and heterosis for quality and rheological traits as well as baking volume

**DOI:** 10.1007/s00122-022-04039-6

**Published:** 2022-02-03

**Authors:** Lea Schwarzwälder, Patrick Thorwarth, Yusheng Zhao, Jochen Christoph Reif, C. Friedrich H. Longin

**Affiliations:** 1 State Plant Breeding Institute, University of Hohenheim, Fruwirthstr. 21, 70599 Stuttgart, Germany; 2 Senior Research Lead Biostatistics and Data Science, KWS Saat SE & Co. KGaA, Grimsehlstr. 31, 37574 Einbeck, Germany; 3 Department of Breeding Research, Leibniz Institute of Plant Genetics and Crop Plant Research (IPK), Corrensstr. 3, 06466 Gatersleben, Germany

## Abstract

**Key message:**

Heterosis effects for dough quality and baking volume were close to zero. However, hybrids have a higher grain yield at a given level of bread making quality compared to their parental lines.

**Abstract:**

Bread wheat cultivars have been selected according to numerous quality traits to fulfill the requirements of the bread making industry. These include beside protein content and quality also rheological traits and baking volume. We evaluated 35 male and 73 female lines and 119 of their single-cross hybrids at three different locations for grain yield, protein content, sedimentation value, extensograph traits and baking volume. No significant differences (*p* < 0.05) were found in the mean comparisons of males, females and hybrids, except for higher grain yield and lower protein content in the hybrids. Mid-parent and better-parent heterosis values were close to zero and slightly negative, respectively, for baking volume and extensograph traits. However, the majority of heterosis values resulted in the finding that hybrids had higher grain yield than lines for a given level of baking volume, sedimentation value or energy value of extensograph. Due to the high correlation with the mid-parent values (*r* > 0.70), an initial prediction of hybrid performance based on line per se performance for protein content, sedimentation value, most traits of the extensograph and baking volume is possible. The low variance due to specific combining ability effects for most quality traits points toward an additive gene action requires quality selection within both heterotic groups. Consequently, hybrid wheat can combine high grain yield with high bread making quality. However, the future use of wheat hybrids strongly depends on the establishment of a cost-efficient and reliable seed production system.

**Supplementary Information:**

The online version contains supplementary material available at 10.1007/s00122-022-04039-6.

## Introduction

Bread wheat varieties *(Triticum aestivum spp. aestivum)* have to fulfill numerous requirements along the supply chain (Thorwarth et al. 2018). Besides high grain yield and disease resistance, which are important for farmers, bread making quality is of great relevance (Oury and Godin 2007). While grain yield can be measured with combine harvesters, good bread making quality encompasses a wide range of properties with time-consuming test methods (Thanhaeuser et al. 2014). Therefore, protein content is often used to predict wheat baking quality because it is quick and easy to measure and serves as the basis for the farmer payment system in many countries (Thorwarth et al. 2018; Boeven and Longin 2019). However, the correlation of protein content with baking volume widely differs across studies (Graybosch et al. 1996; Uhlen et al. 2004; Koppel and Ingver 2010; Maphosa et al. 2015). Furthermore, the negative correlation of protein content and grain yield requires high amounts of nitrogen fertilizers to realize high grain yields with acceptable protein contents with negative impact on the environment (Zörb et al. 2018). Therefore, more information about dough rheological traits and baking volume needs to be considered (Koppel and Ingver 2010).

Brabender extensograph is a standard method to investigate dough rheological properties such as dough extensibility and elasticity, which provides insights into important processing properties (Frakolaki et al. 2018). In addition to dough properties, shape and volume of the final product, other quality aspects such as storage potential and freshness are also relevant for the product quality (Freund and Kim 2006). High water absorption promotes a good baking volume, good freshness of the end product and improved storage potential (Puhr and D’Appolonia 1992; Koppel and Ingver 2010). It is also of practical interest because with a high water absorption, less flour is required to reach a certain loaf volume (Koppel and Ingver 2010; Frakolaki et al. 2018).

In the last decade, hybrid wheat breeding gained much interest in the public and private sector (Boeven and Longin 2019). Hybrid breeding is well established in many outcrossing species but is still under development in wheat (Gupta et al. 2019). A mid-parent heterosis for grain yield of approximately 10% has been reported for hybrid bread and durum wheat (Gowda et al. 2010; Thorwarth et al. 2018). For protein content, negative heterosis values were reported, which might be explained by the negative correlation between grain yield and protein content (Oury and Godin 2007; Thorwarth et al. 2018, 2019; Boeven and Longin 2019). However, some recent studies have shown that hybrid bread and durum wheat can combine good sedimentation values, acceptable protein content with high grain yield (Thorwarth et al. 2018; Akel et al. 2019; Boeven and Longin 2019). To the best of our knowledge, no study has investigated other bread making quality traits like extensograph traits and baking volume at a considerably high number of bread wheat hybrids and their parental lines.

We therefore investigated 35 male and 73 female lines and 119 of their single-cross hybrids at three different locations for grain yield, dough quality and baking volume. Our objectives were to (1) evaluate variance components, trait correlations and heritabilities for the examined quality traits, (2) estimate the extend of mid- and better-parent heterosis, (3) assess the association between mid-parent value, line per se performance and GCA effects with hybrid performance and (4) evaluate the potential of hybrids to combine good bread making quality with high grain yield.

## Materials and methods

### Plant material and field experiments

The initial study was based on phenotypic data of 236 elite winter bread wheat lines (Triticum aestivum ssp. aestivum) used as parents representing Central European diversity and their 1744 single-cross hybrid progenies. Lines were distinguished into two groups of 40 males and 196 females, considering pollination ability, flowering time and plant height. Hybrids were crossed in an incomplete factorial mating design. Additionally, 11 check varieties were included (Colonia, Elixer, Hystar, Hybred, JBAsano, Julius, KWSLoft, LGAlpha, RGTReform, Rumor and Tobak). The initial experimental setup was described by Zhao et al. (2021). Due to capacity limits in the baking quality analytics, the presented data were based on a selected fraction of the initial plant material of 35 male and 73 female lines and 119 of their single-cross hybrids depending on different criteria such as varying line per se and hybrid performance for agronomic traits, protein content and sedimentation value or diversity in glutenin bands. This selection led to a representative subsample of the initial 1744 hybrids as reflected by a PCA (Principal Component Analysis) based on the BLUEs (Best Linear Unbiased Estimates) for grain yield, protein content and sedimentation value (Suppl. Fig S2). Furthermore, with that selection almost half of the females and males were represented in ≥ 2 and ≥ 3 hybrid combinations (Suppl. Table 1). Samples of the selected fraction were taken from the location in Hadmersleben (51°59'31''N, 11°18'5''E, Germany, mean temp. 11.2 °C and precipitation 334.2 mm in 2018), Seligenstadt (50°3'N, 8°59'E, Germany, mean temp. 11 °C and precipitation 470.8 mm in 2018) and Gola (51°49'16.2'N 16°52'32.7''E, Poland, mean temp. 10.3 and precipitation 781.8 mm in 2018). Experiments were conducted during the growing season 2018. In each environment, the experimental design consisted of three trials. In each trial, an un-replicated alpha lattice design was used.

Different genotypes were evaluated in different trials linked by the 11 common checks. Plot size ranged between 5.70 and 9 m^2^. All plots were treated with fertilizer, pesticides and fungicides according to farmers’ practice for intensive wheat production.

Grain yield was measured in tons per hectare (t/ha) with an adjusted moisture content of 14%. Protein content (%) was determined with a near-infrared reflectance (NIR) spectrometry (ICC standard method 159, ICC, Vienna, Austria). Wet gluten content (%) was measured with Perten Glutomatic (ICC standard method 137/1, ICC, Vienna, Austria). Sedimentation value was determined according to Zeleny (ICC standard method 116/1, ICC, Vienna, Austria). Five quality traits were assessed with the Brabender extensograph (ICC standard method Nr. 114/1, ICC, Vienna, Austria) but with only one measurement instead of two for each trait. Traits are extensibility (mm), resistance to extension (EU), the ratio between extensibility and resistance to extension, energy (cm^2^) as the area under the extensograph curve and water absorption (%) of the dough. Maltose (%) was determined after the Berlin method (Klüver 1994). Browning was visually scored from 1 (high intensity) to 5 (low intensity) by an experienced cereal scientist (Quality Lab Aberham, Augsburg, Germany). Baking volume (ml) was determined with a rapid method adapted from Rapid Mix test (ICC standard method 131, ICC, Vienna, Austria) but with lower amount of flour and test bread rolls (Quality Lab Aberham, Augsburg, Germany). All traits and abbreviations are summarized in Table 1.

**Table 1:** Agronomic and quality traits assessed in the study

Trait	Abbreviation	Unit of measurement
Grain yield	GY	t/ha
Protein content	PC	%
Wet gluten content	WGC	%
Sedimentation value	SDS	ml
Extensibility	EX	mm
Resistance to extension	REX	EU
REX/EX Ratio	R	EU /mm
Energy	EN	cm^2^
Water absorption	WA	%
Maltose	MT	%
Browning	BR	1 = high, 5 = low
Baking volume	BV	ml

### Phenotypic data analyses

As suggested by Bernal-Vasquez et al. (2016) an outlier detection was performed before analyzing the phenotypic data following the method 4 ‘‘Bonferroni-Holm with re-scaled median absolute deviation standardized residuals.” The best linear unbiased estimators (BLUEs) were then calculated based on the following mixed model (1):

$$y_{ikl} = \mu + g_{i} + e_{l} + t_{kl} + \left( {ge} \right)_{il} + {\mathcal{E}}_{ikl} ,$$

where ${y}_{ikl}$ is the phenotypic observation of the *i*th genotype within the *k*th trial at the *l*th environment. The intercept is denoted as $\mu$, ${g}_{i}$ is the effect of the *i*th genotype, ${e}_{l}$ the effect of the *l*th environment, where environment is defined as location times year combination, ${t}_{kl}$ is the effect of the *k*th trial at the *l*th environment, ${(ge)}_{il}$ represents the interaction effect between genotype and environment, and ${\mathcal{E}}_{ikl}$ is the residual term of ${y}_{ikl}$. For the calculation of the BLUEs all effects were taken as random, except ${g}_{i}$. The error variance was assumed to be heterogeneous.

With an extended model (2), we dissected genetic variance into variances for general (GCA) and specific combining ability (SCA) to evaluate GCA, SCA and heterotic effects (Beukert et al. 2020):

$$y_{ijkl} = \mu + e_{l} + t_{kl} + g_{ij} + m_{i} + f_{j} + s_{ij} + \left( {me} \right)_{il} + \left( {fe} \right)_{jl} + {\mathcal{E}}_{ijkl} ,$$

${y}_{ijkl}$ is the phenotypic observation of lines $(i=j)$ or hybrids $(i\ne j)$, where hybrids were denoted as a cross between the *i*th parent with the *j*th parent. $\mu$ is the the overall population mean, ${e}_{l}$ and ${t}_{kl}$ follow the same notation as in model (1). ${g}_{ij}$ refers to the genotypic effect of parental lines, ${m}_{i}$ is the GCA effect of the *i*th male parent, ${f}_{j}$ the GCA effect of the *j*th female parent and ${s}_{ij}$ represents the SCA effect of the cross between the *i*th male with the *j*th female. Interaction effects between GCA effects of the *i*th male and the *j*th female with the *l*th environment were modeled as ${(me)}_{il}$ and ${(fe)}_{jl}$. ${\mathcal{E}}_{ijkl}$ is the residual term of ${y}_{ijkl}$. All effects were modeled as random effects except $\mu$ and the error variance was assumed to be heterogeneous for each environment. For simplicity of illustration, however, the average of these error variances is shown in Table 2. Significance of variance components were based on their z-ratios.

**Table 2:** Estimates of variance components, heritabilities, mean and range of mid-parent (MPH) and better-parent (BPH) heterosis, correlations between mid-parent values and hybrid performance r(MP, HYB), general combining ability effects and hybrid performance r(GCA, HYB) as well as general combining ability effects and line per se performance r(GCA, per se)

Source	GY (t/ha)	PC (%)	WGC (%)	SDS (ml)	EX (mm)	REX (EU)	R (REX/EX)	EN (cm^2^)	WA (%)	MT (%)	BR (1–5)	BV (ml)
*Parents*												
${\upsigma }_{\mathrm{G}}^{2}$	9.44***	0.48***	2.54***	89.89***	204.01***	2793.56***	0.10***	851.88***	4.82***	0.02***	0.26***	3007.55***
${\upsigma }_{\mathrm{GxE}}^{2}$	11.49***	0.12**	1.24	12.80***	0.00	0.00	0.02	0.00	0.27***	0.00	0.04	0.00
${\mathrm{h}}^{2}$	0.45	0.85	0.76	0.89	0.91	0.93	0.90	0.94	0.97	0.77	0.72	0.93
${F}_{1}$*-hybrids*												
${\upsigma }_{\mathrm{G}}^{2}$	0.19	0.17***	1.33***	59.06***	87.80***	1868.73***	0.06***	483.99***	2.42***	0.01***	0.11***	2083.50***
${\upsigma }_{\mathrm{GCA}-\mathrm{F}}^{2}$	5.88	0.23***	1.60***	44.91***	91.19***	1196.00***	0.04***	391.30***	1.66***	0.01***	0.12*	1718.82***
${\upsigma }_{\mathrm{GCA}-\mathrm{M}}^{2}$	3.47	0.06	0.46*	19.04*	22.26*	583.85***	0.02**	108.77***	0.37**	0.0001	0.01	415.64**
${\upsigma }_{\mathrm{SCA}}^{2}$	0.15	0.00	0.15	8.98*	1.04	96.81	0.0037	0.00	0.21**	0.0012	0.05	64.68
${\upsigma }_{\mathrm{GCA}-\mathrm{FxE}}^{2}$	10.06**	0.01	0.54*	6.10*	15.64	64.91	0.0027	20.02	0.14**	0.0022	0.09**	76.89
${\upsigma }_{\mathrm{GCA}-\mathrm{MxE}}^{2}$	4.92	0.01	0.03	1.99	0.41	66.70	0.0025	15.08	0.03	0.0006	0.00	22.67
${\upsigma }_{\mathrm{e}}^{2}$	28.65	0.32	1.74	12.89	57.75	489.76	0.03	120.78	0.31	0.01	0.26	701.62
${\mathrm{h}}^{2}$	0.43	0.75	0.74	0.86	0.86	0.89	0.85	0.90	0.93	0.64	0.63	0.90
${\upsigma }_{\mathrm{SCA}}^{2}$/${\upsigma }_{\mathrm{GCA}}^{2}$	0.02	0.00	0.07	0.12	0.01	0.05	0.06	0.00	0.09	0.12	0.29	0.03
MPH (%)	6.36	− 1.66	− 1.29	0.57	1.04	1.62	2.63	1.52	− 0.62	− 0.91	4.22	0.67
Range MPH (%)	− 14.81 to 7.37	− 10.50 to 10.16	− 13.07 to 30.05	− 34.59 to 39.04	− 14.27 to 22.46	− 30.94 to 45.98	− 40.21 to 66.20	− 32.50 to 56.95	− 5.81 to 6.03	− 14.10 to 12.57	− 42.09 to 67.08	− 10.83 to 19.13
BPH (%)	3.40	− 4.94	− 4.59	− 11.25	− 4.76	− 10.18	− 8.34	− 15.34	− 3.14	− 5.17	− 8.94	− 4.15
Range BPH (%)	− 19.93 to 17.15	− 18.83 to 6.26	− 20.38 to 27.70	− 51.91 to 38.33	− 17.34 to 22.39	− 44.00 to 30.65	− 43.02 to 47.25	−56.31–39.65	− 10.43 to 4.69	− 22.39 to 11.32	− 53.60 to 43.48	− 18.33 to 14.47
r(MP, HYB)	0.42***	0.71***	0.49***	0.81***	0.59***	0.67***	0.48***	0.84***	0.76***	0.65***	0.55***	0.84***
r(GCA, HYB)	0.94***	0.95***	0.96***	0.97***	0.96***	0.98***	0.97***	0.99***	0.98***	0.91***	0.90***	0.99***
r(GCA, per se)	0.38***	0.62***	0.42***	0.75***	0.53***	0.65***	0.53***	0.73***	0.74***	0.60***	0.51***	0.77***

For abbreviations of the traits, see Table 1

${\upsigma }_{\mathrm{G}}^{2}$ = Genetic variance, ${\upsigma }_{\mathrm{e}}^{2}$ = Error variance, ${\upsigma }_{\mathrm{GxE}}^{2}$ = Variance of genotype-by-environment interaction, ${\upsigma }_{\mathrm{GCA}-\mathrm{F}}^{2}$ = GCA variance of females, ${\upsigma }_{\mathrm{GCA}-\mathrm{M}}^{2}$ = GCA variance of males, ${\upsigma }_{\mathrm{SCA}}^{2}$ = SCA variance, ${\upsigma }_{\mathrm{GCA}-\mathrm{FxE}}^{2}$ = Variance of female GCA-by-environment interaction, ${\upsigma }_{\mathrm{GCA}-\mathrm{MxE}}^{2}$ = Variance of male GCA-by-environment interaction, ${\upsigma }_{\mathrm{SCA}}^{2}$/${\upsigma }_{\mathrm{GCA}}^{2}$ = Ratio of SCA variance and the sum of GCA female, GCA male and SCA variance (${\upsigma }_{\mathrm{GCA}}^{2}$), ${\mathrm{h}}^{2}$ = Heritability

*, **, ***Significantly different from zero at the 0.05, 0.01 and 0.001 level of probability, respectively

Broad-sense heritability was computed separately for hybrids and lines following Piepho and Möhring (2007):

$$H^{2} = \frac{{\delta_{g}^{2} }}{{\delta_{g}^{2} + \frac{v}{2}}} ,$$

where $\frac{v}{2}$ is the mean variance of a difference of two adjusted treatment means and ${\delta }_{g}^{2}$ the genetic variance estimated with model (2). The mid-parent heterosis (MPH) was calculated as MPH = HYB–MP and better-parent heterosis as BPH = HYB–${\mathrm{P}}_{\mathrm{max}}$, where ${\mathrm{P}}_{\mathrm{max}}$ is the performance of the better-parent and HYB the performance of hybrids. MP is the mid-parent performance, determined as MP = (${P}_{1}$+${P}_{2}$)/2, where ${P}_{1}$ and ${P}_{2}$ are the performances of parents of specific hybrids. In addition, we calculated the Pearson’s correlation coefficient of MP with HYB r = (MP, HYB), the sum of GCA effects with HYB r = (GCA, HYB) and line per se performance r = (GCA, per se). We also calculated the Pearson’s correlation coefficients of phenotypic values among all traits.

All analyses were performed within the R software (R Core Team 2021) and the software ASReml 3.0 (Gilmour et al. 2009).

## Results

The 109 parental lines and their 119 hybrids varied largely in all traits considered, resulting in highly significant genetic variances for all traits (Table 2). The sum of GCA variances were considerably smaller than the genetic variance within the parental lines except for grain yield. For instance, for baking volume, the genetic variance for parental lines was 3007.55 ml^2^ (*p* < 0.01) but the sum of GCA variances in their hybrids only 2134.46 ml^2^. Thus, the exploitable variance for selection is higher for line than for hybrid breeding. The GCA variance for male lines was lower than that for female lines across all traits, which may be due to the fact that we used only 35 male lines but 74 female lines in our study. The ${\upsigma }_{\mathrm{SCA}}^{2}$ /${\upsigma }_{\mathrm{GCA}}^{2}$ ratio was low for all traits ranging from 0 for energy value of extensograph to 0.29 for browning of the bread crust.

Estimates of variance components have large standard errors, the larger the lower the trait heritability and the larger the complexity of variance components, e.g., SCA versus GCA. For instance, for baking volume, standard error for SCA variance was 127% in relation to SCA variance while for GCA it was 20% and 37% for males and females, respectively (data not shown). For grain yield with considerably lower heritability, it was more pronounced with a standard error of the SCA variance of 2.21 (data not shown). Thus, we speculate that for grain yield the SCA variance in our study is underestimated as reflected by larger SCA variances reported in the literature based on larger numbers of hybrids and locations (Oettler et al. 2005; Longin et al. 2013; Miedaner et al. 2017; Zhao et al. 2021). Thus, we concentrate our further discussion on variance components of quality traits.

With the exception for grain yield, genotype-by-environment interaction variances were considerably lower than genetic variances resulting in high heritability estimates for all traits. Furthermore, heritability estimates were comparable for parental lines and hybrids with a tendency toward slightly lower values for hybrids for few traits.

**Fig. 1:**
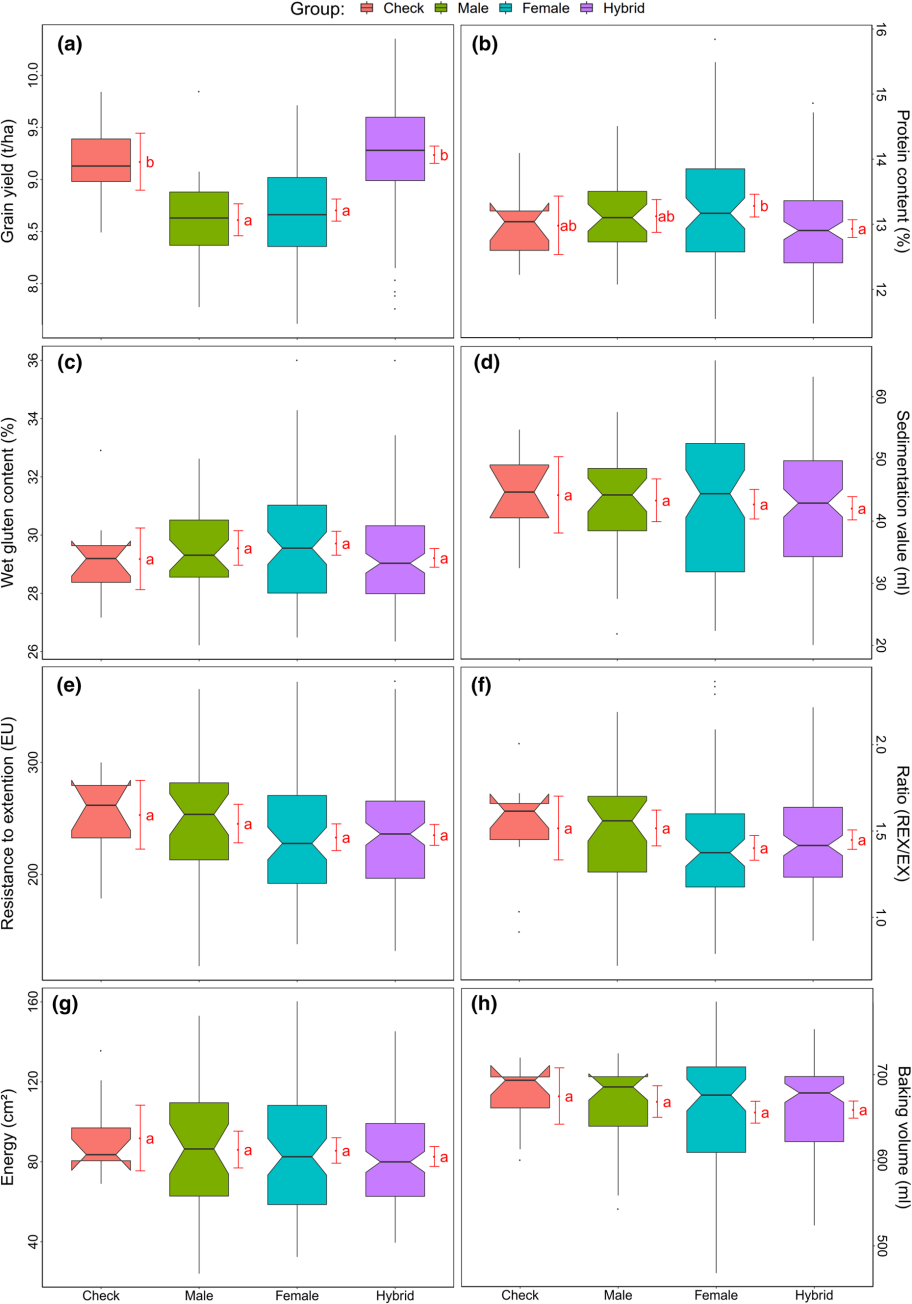
Boxplots for different traits grouped by checks, males, females and hybrids. Means between groups with a common letter for a given trait do not differ significantly from each other based on Tukey’s test

The average mid-parent heterosis was positive for grain yield, sedimentation value, all dough traits measured with extensograph (EX, REX, R, EN), browning of the bread crust, and baking volume (Table 2), while it was negative for the remaining traits. Regarding average better-parent heterosis, positive values were determined only for grain yield. Nevertheless, a wide range of trait values were found within the groups of male and female lines as well as hybrids across all quality traits (Fig. 1). Differences between the groups of males, females, checks and hybrids were therefore not significant for all quality traits except for protein content, which was slightly higher in parental lines than in hybrids. It is important to note that quality analysis of hybrids is based on F_2_-kernels, i.e., the harvest of the F_1_-hybrids, while agronomic traits were measured at the F_1_-plants. Nevertheless, millers and bakers will also get the harvest of the F_1_-hybrids, i.e., F_2_-kernels and consequently, our quality analyses reflect practice. The correlation of mid-parent and hybrid performance varied largely from 0.42 (*p* < 0.01) for grain yield up to 0.84 (*p* < 0.01) for energy value of extensograph and baking volume (Table 2). The correlation of GCA and hybrid performance was larger than the correlation of mid-parent and hybrid performance for all traits.

Grain yield was moderately negatively correlated with all quality traits except for maltose content and browning of the bread crust (Table 3). Thereby, the correlation coefficients for the parental lines and hybrids were similar in magnitude. Baking volume correlated highest with sedimentation value at 0.89 (*p* < 0.01) for the lines and hybrids. The second highest correlation to baking volume was recorded for the energy value of extensograph with 0.83 (*p* < 0.01) and 0.87 (*p* < 0.01) for the lines and hybrids, respectively. In contrast, the correlation coefficients between baking volume and protein or gluten content was considerably lower.

**Table 3:** Phenotypic correlation coefficients among 12 traits determined either for 119 F_1_-hybrids (above diagonal) or for inbred lines (below diagonal; consisting of 35 male lines, 73 female lines and 11 checks)

Parents/F_1_-hybrids	GY	PC	WGC	SDS	EX	REX	R	EN	WA	MT	BR	BV
GY		− 0.45***	− 0.45***	− 0.36***	− 0.42***	− 0.20*	− 0.10	− 0.34***	− 0.21*	0.03	0.08	− 0.38***
PC	− 0.51***		0.76***	0.69***	0.49***	0.41***	0.28**	0.58***	0.59***	− 0.37***	− 0.40***	0.72***
WGC	− 0.43***	0.86***		0.54***	0.48***	0.18*	0.03	0.41***	0.56***	− 0.24**	− 0.27**	0.59***
SDS	− 0.34***	0.61***	0.54***		0.63***	0.63***	0.44***	0.81***	0.66***	− 0.50***	− 0.50***	0.89***
EX	− 0.09	0.40***	0.46***	0.51***		0.22*	− 0.08	0.59***	0.40***	− 0.15	− 0.20*	0.66***
REX	− 0.21*	0.27**	0.13	0.68***	0.24**		0.94***	0.88***	0.21*	− 0.26**	− 0.31***	0.70***
R	− 0.18*	0.16	− 0.03	0.51***	− 0.10	0.92***		0.70***	0.10	− 0.22*	− 0.25**	0.51***
EN	− 0.22*	0.41***	0.35***	0.80***	0.60***	0.88***	0.67***		0.38***	− 0.32***	− 0.37***	0.87***
WA	− 0.23*	0.50***	0.64***	0.48***	0.18*	0.06	0.00	0.18*		− 0.62***	− 0.57***	0.57***
MT	0.10	− 0.20*	− 0.30***	− 0.38***	− 0.24**	− 0.09	− 0.01	− 0.20*	− 0.67***		0.92***	− 0.48***
BR	0.15	− 0.22*	− 0.35***	− 0.43***	− 0.31***	− 0.10	0.01	− 0.25**	− 0.69***	0.91***		− 0.51***
BV	− 0.28**	0.59***	0.59***	0.89***	0.62***	0.70***	0.49***	0.83***	0.44***	− 0.37***	− 0.45***	

For abbreviations of the traits, see Table 1

*, **, ***Significantly different from zero at the 0.05, 0.01 and 0.001 level of probability, respectively

**Fig. 2:**
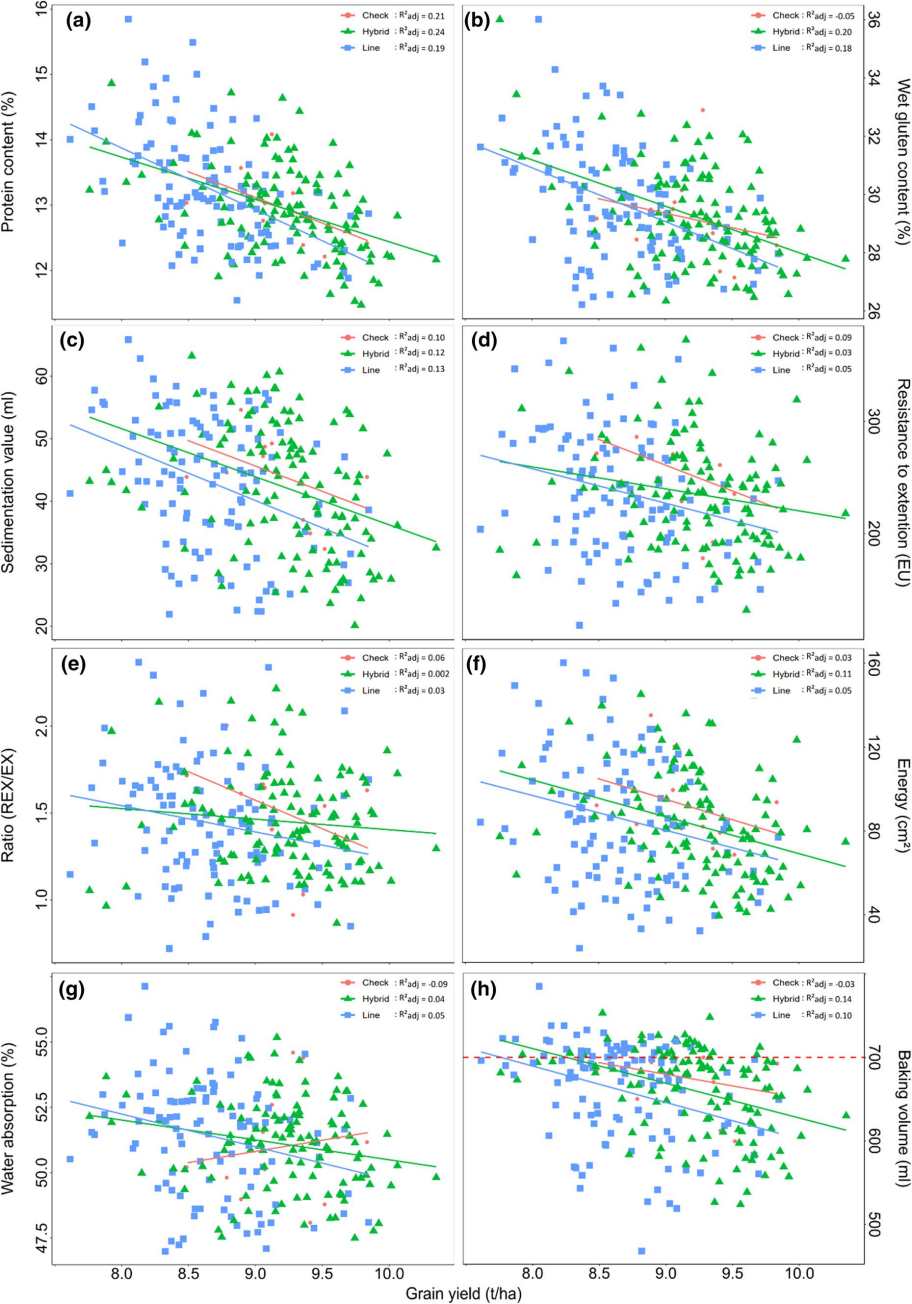
Linear regression plots of **a** protein content, **b** wet gluten content, **c** sedimentation value, **d** resistance to extension, **e** ratio of REX/EX, **f** energy, **g** water absorption and **h** baking volume on grain yield. The regression line of check varieties is colored in red, of hybrids in green and of lines in blue. * R*^2^_adj_ represents the adjusted * R*^2^ for the regression lines of checks, hybrids and lines, respectively (color figure online)

**Fig. 3:**
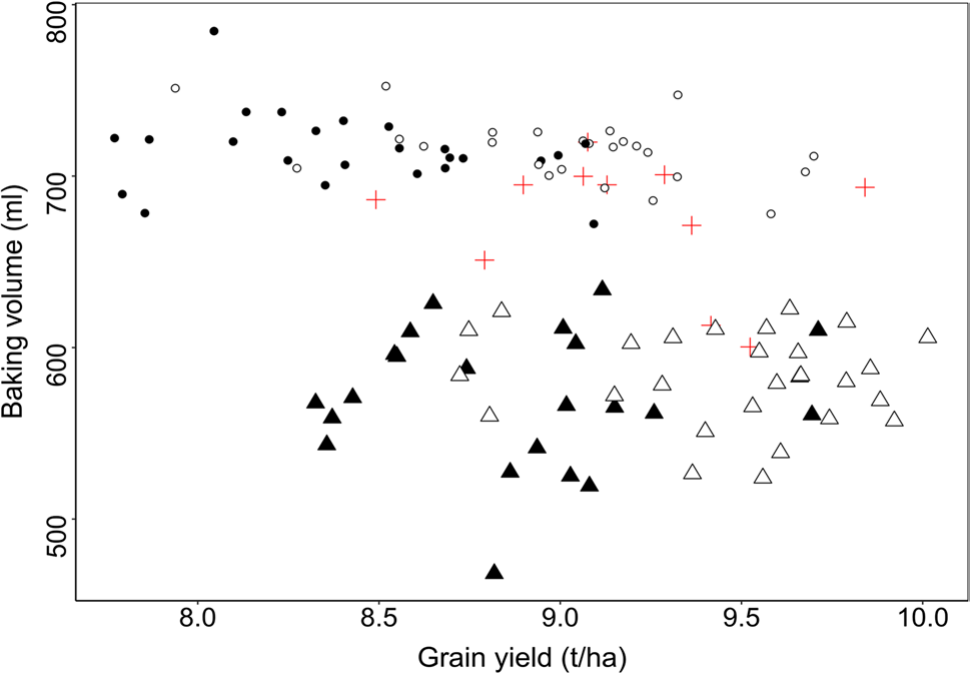
Baking volume plotted against grain yield of lines (filled symbols) and hybrids (empty symbols) belonging to the 20% best (circles), 20% worst (triangles) genotypes regarding sedimentation value and check varieties (red crosses) (color figure online)

The negative correlation of grain yield with many quality traits was also visible when quality traits were plotted against grain yield (Fig. 2). The large variation in parental lines and hybrids within the individual traits, however, allows for selection of “outliers” from that negative correlation having high yield and good quality. For instance, considering a baking volume greater than 700 ml (Fig. 2h), there was still a wide variation in grain yield available for selection ranging from 7.7 to 9.7 t/ha. Interestingly, the best line in that quality class had a yield of only 9.2 t/ha, showing an advantage of hybrids of 0.5 t/ha. This advantage of hybrids was also confirmed when a selection step was applied to sedimentation value taking either the 20% best or worst genotypes before plotting their baking volumes against grain yield (Fig. 3).

## Discussion

Contrary to previous assumptions, several studies showed that hybrid bread and durum wheat can combine good sedimentation values, acceptable protein content with high grain yield (Thorwarth et al. 2018; Akel et al. 2019; Boeven and Longin 2019). However, more information is required for bread making in terms of dough properties and baking volume, which is to the best of our knowledge not yet investigated on a larger number of hybrids and parental lines.

### Hybrid wheat can have good baking quality

We observed an average mid-parent heterosis for baking volume of 0.67% (Table 2). Average better-parent heterosis (BPH) was −4.15% but ranged up to 14.47%. Furthermore, no significant difference in baking volume was observed between the groups of male and female lines, check varieties as well as hybrids (Fig. 1h). Similarly, within the ten genotypes with highest baking volume we found six parental lines and four hybrids (data not shown). Thus, a hybrid wheat variety can have a baking volume similar to the best quality line variety.

Even more, hybrid wheat has on average a higher grain yield at a given level of baking volume. For instance, assuming a baking volume of 700 ml we found only two parental lines with grain yield higher than 9 t/ha surrounded by plenty of hybrids (Fig. 2h). Similarly, the widely grown line variety RGTReform had a baking volume of 719.8 ml and a grain yield of 9.0 t/ha in our trial. We found one hybrid with ~ 3% higher grain yield and slightly better baking volume but no parental line with better grain yield at the given quality level. Similar results were found for sedimentation value or dough traits measured by the extensograph. Average heterosis values were negative but ranged widely up into positive values (Table 2) leading to yield advantages of hybrids at given levels of sedimentation values or extensograph traits (Fig. 2). This confirms previous studies on sedimentation value in durum and bread wheat (Thorwarth et al. 2018; Akel et al. 2019). No comparable literature was available for heterotic effects of extensograph traits or baking volume.

By contrast, average mid-parent heterosis was negative for protein content and wet gluten content (Table 2). Although a wide range in heterosis values was visible for both traits, genotypes with the highest protein or wet gluten content belonged mainly to the group of lines (Fig. 2a, b). This confirms previous studies on protein content of durum and bread wheat (Gowda et al. 2010; Thorwarth et al. 2018) and might be explained by the negative correlation of grain yield and protein content (Table 3) leading in tendency to low protein contents in genotypes with high grain yield.

Similarly, average mid- and better-parent heterosis was negative for the trait water absorption (Table 2) and genotypes with the highest water absorption predominantly belonged to the group of lines (Fig. 2g). Water absorption correlated positively with protein and wet gluten content in lines and hybrids, respectively (Table 3). Thus, the negative average heterosis might be explained by the amount of gluten, which is reported to be partly responsible for water absorption in wheat dough (Wieser 2007; Kaushik et al. 2015). Water absorption is also influenced by kernel hardiness, which is a trait influenced by few major and many minor genes (Mikulikova 2007; Mohler et al. 2012). However, we did not investigate kernel hardiness requiring further research for quality breeding in hybrids.

Ignoring very high values of protein content (> 14%), wet gluten content (> 32%) and water absorption (> 55%) led to the same observation as discussed above for sedimentation value or baking volume: At a given quality trait level, hybrids have almost always a higher grain yield than their parental lines (Fig. 2). For instance, taking the actually most popular wheat variety in Germany RGTReform as reference, we found hybrids which had similar or better baking volume, sedimentation value, protein content and wet gluten content but around 4% higher grain yield. Summarizing, hybrid wheat can have high bread making quality expressed in baking volume, sedimentation value or dough properties at acceptable levels of protein and wet gluten content but at higher grain yield compared with line varieties.

### How to breed hybrids with high baking quality?

We determined a low ratio of ${\upsigma }_{\mathrm{SCA}}^{2}$ /${\upsigma }_{\mathrm{GCA}}^{2}$ = 0.03 for baking volume (Table 2) which points toward a mainly additive gene action of that trait. Furthermore, the correlation of mid-parent and hybrid performance was high with *r* = 0.84 (*p* < 0.01). Similar results were obtained for sedimentation value, water absorption and the energy value of the extensograph. Consequently, high-quality parental lines should be chosen in both heterotic groups to maximize baking quality of hybrids.

In contrast to our results, Oettler et al. (2005), Longin et al. (2013), Miedaner et al. (2017), Thorwarth et al. (2018) and Zhao et al. (2021) reported higher amounts of SCA and lower correlation coefficients between mid-parent and hybrid performance for grain yield or resistance to some diseases. Consequently, these authors recommended to predict hybrids based on GCA values rather than using parental line per se performance values thereby making hybrid breeding in wheat expensive and slow as compared to line breeding (Boeven and Longin 2019). By contrast, our findings on a high correlation between mid-parent and hybrid performance for most important wheat quality traits enables selection of parental lines based on their per se values largely facilitating breeding for high bread making quality in hybrid wheat.

Correlation coefficients between grain yield and quality traits as well as between quality traits were of similar magnitude in hybrids and lines (Table 3). For instance, baking volume correlated highest with sedimentation value and the energy value of extensograph while considerably lower with protein and wet gluten content in lines and hybrids. Similarly, protein content, wet gluten content, sedimentation value and baking volume were moderately negatively correlated with grain yield in parental lines and hybrids. Thus, knowledge collected across decades on quality selection in line breeding could be directly applied in hybrid wheat breeding.

### Breeding for high-quality hybrids

The mainly additive gene action for most important quality traits discussed above requests for quality breeding in both heterotic groups. Selection for the important trait baking volume cannot be performed in early generations, as testing needs a lot of grains, requires standardized milling and baking and is expensive and slow. Sedimentation value has a high heritability, a high correlation to baking volume, only few grams of flour are required, several hundred tests can be run within a day and the correlation between mid-parent and hybrid performance is high making it interesting as important trait to be measured as soon as possible in early generations. To validate its use, we selected the 20% best and worst lines and hybrids in sedimentation value and plotted their baking volume against their grain yield (Fig. 3).

To our opinion, three important points became visible. First, the groups selected for sedimentation value led to a clear separation of these groups regarding baking volume; thus, a selection on sedimentation value is effective for improving baking volume. Second, irrespective of the selected group, hybrids had a higher grain yield at a given quality level underlining our findings discussed above. And third, grain yield in lines and hybrids was higher in the group with low sedimentation value and baking volume. Selection on quality traits might therefore lead to reduced yield potential, which can be explained by the negative correlation between grain yield and most quality traits. Thus, in early generations, the breeder has to compromise between selection for the required quality traits and selection to maintain or even improve a high yield level.

In later breeding generations, testing on baking volume is required as even correlation coefficients between sedimentation value and baking volume of around 0.90 can lead to misclassifications in that important trait. Furthermore, the correlation between GCA and hybrid performance is considerably higher than the correlation between mid-parent and hybrid performance for baking volume (Table 2, Suppl. Figure 1). Thus, selection within heterotic groups should be based on GCA values as soon as they become available, latest after first yield tests of new parental lines. As for grain yield the correlation of mid-parent and hybrid performance is low (Table 2), efficient hybrid breeding on grain yield requires GCA values as soon as possible in the breeding program. This also enables to estimate GCA values for baking volume at least for agronomically promising new parental lines.

Application of genomic selection has shown to be very promising to facilitate hybrid wheat breeding in agronomic traits (Zhao et al. 2013, 2014; Basnet et al. 2019). In line breeding, genomic selection was already shown to be of high interest especially in quality selection due to the slow and expensive procedure of baking tests (Battenfield et al. 2016; Michel et al. 2018). Thus, further research is urgently required to evaluate the use of genomic selection for quality breeding in hybrid wheat (Thorwarth et al. 2019).

## Conclusions

We could clearly elaborate that wheat hybrids can have excellent quality comparable to best quality of line varieties. Even more, for a similar level of baking volume, sedimentation value and important dough traits, the highest grain yield was always achieved for hybrids and not for lines in our study although best breeding lines from all German wheat breeding companies were used as parental lines. Only for protein and wet gluten content, lines were slightly better than hybrids but the large variability in these traits allows for selection of hybrids with acceptable amounts of protein and gluten. Baking volume, sedimentation value, energy value from extensograph and water absorption of the dough had high heritabilities, low ${\upsigma }_{\mathrm{SCA}}^{2}$, and high correlations between mid-parent and hybrid performance. This facilitates on the one hand breeding for quality in hybrid wheat as per se values of parental lines could be used as predictors for their GCA and hybrid performance. On the other hand, the almost additive gene action requires quality breeding in both heterotic groups. However, to our opinion, these findings have limited importance on the future effectiveness of hybrid versus line breeding in wheat. This mainly depends on rapid improvements in hybrid seed production technologies and speedup of hybrid programs with genomic selection and other predictive tools.

## Supplementary Information

Below is the link to the electronic supplementary material.

Association of 119 hybrids with (a) the mean of parental lines and (b) mean GCA effects of parental lines for baking volume. R²adj represents the adjusted R² for the regression lines of hybrid performance on mid-parent value and GCA effects of parental lines, respectively.
Supplementary file1 (PDF 261 kb)

Principal Component Analysis (PCA) of grain yield, protein content and sedimentation volume in the full set of 1.744 hybrids (red dots) initially tested in agronomic trials and the subset of the 119 selected hybrids thereof for in-depth quality analyses (blue dots).
Supplementary file2 (PDF 17 kb)

Estimated BLUEs (best linear unbiased estimator) of the evaluated traits as well as females and males and their use in hybrid combinations.
Supplementary file3 (XLSX 62 kb)

Raw values of the three environments of the evaluated traits.
Supplementary file4 (XLSX 73 kb)
