# Supplementary figures and images for: Hybrid wheat: quantitative genetic parameters and heterosis for quality and rheological traits as well as baking volume
Source: Theor Appl Genet. 2022 Feb 3;135(4):1131–41. doi: 10.1007/s00122-022-04039-6 (PMC9033736; doi:10.1007/s00122-022-04039-6)

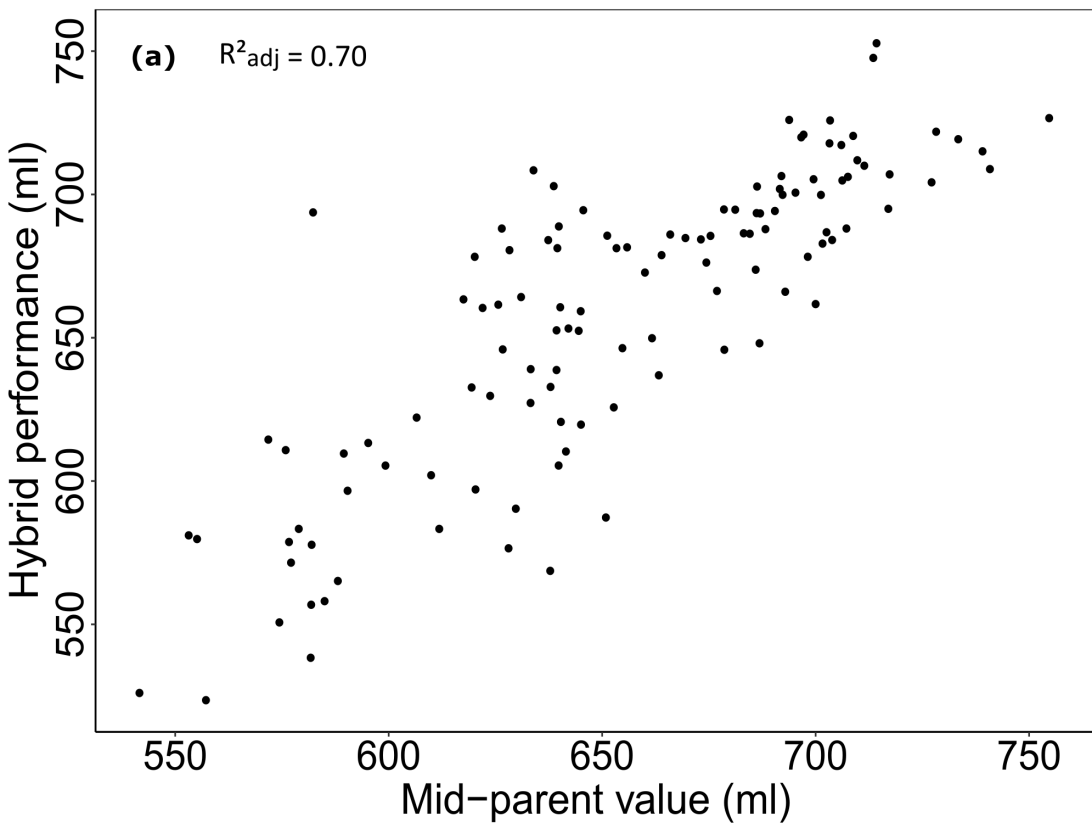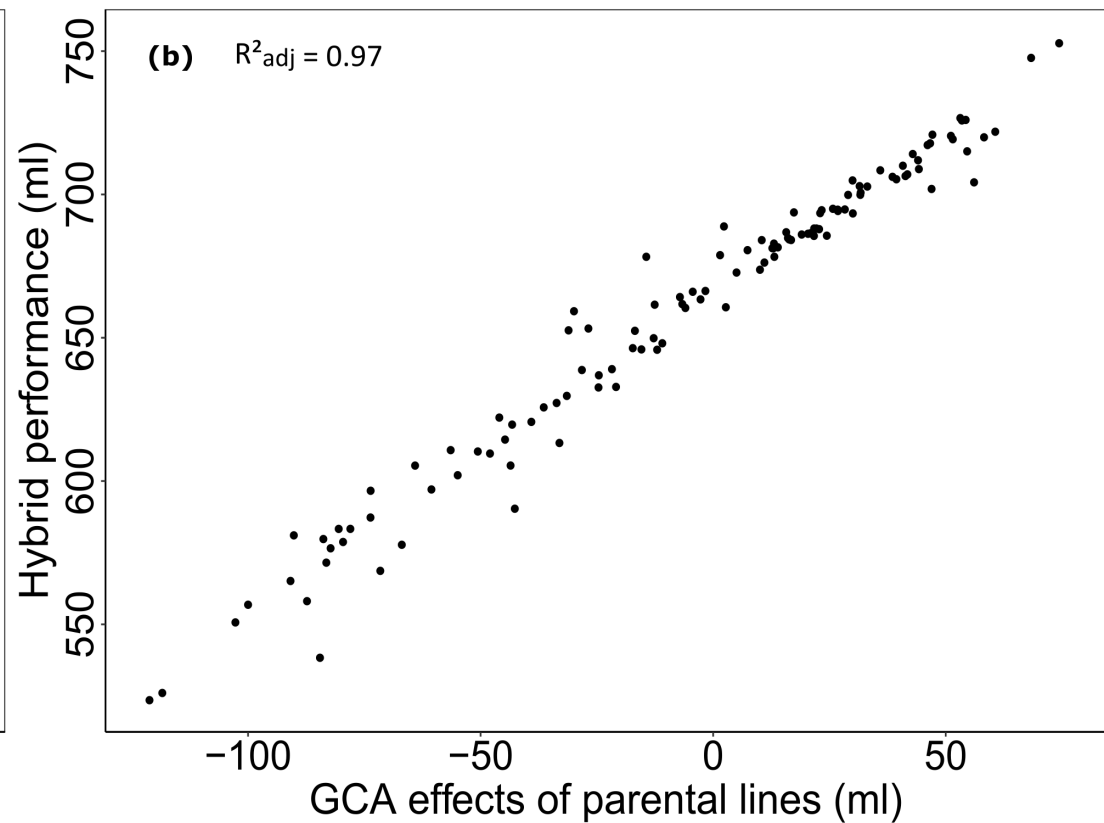

Supplement: Supplementary file 1 — Association of 119 hybrids with (a) the mean of parental lines and (b) mean GCA effects of parental lines for baking volume. R²adj represents the adjusted R² for the regression lines of hybrid performance on mid-parent value and GCA effects of parental lines, respectively. Supplementary file1 (PDF 261 kb) [file 122_2022_4039_MOESM1_ESM.pdf]

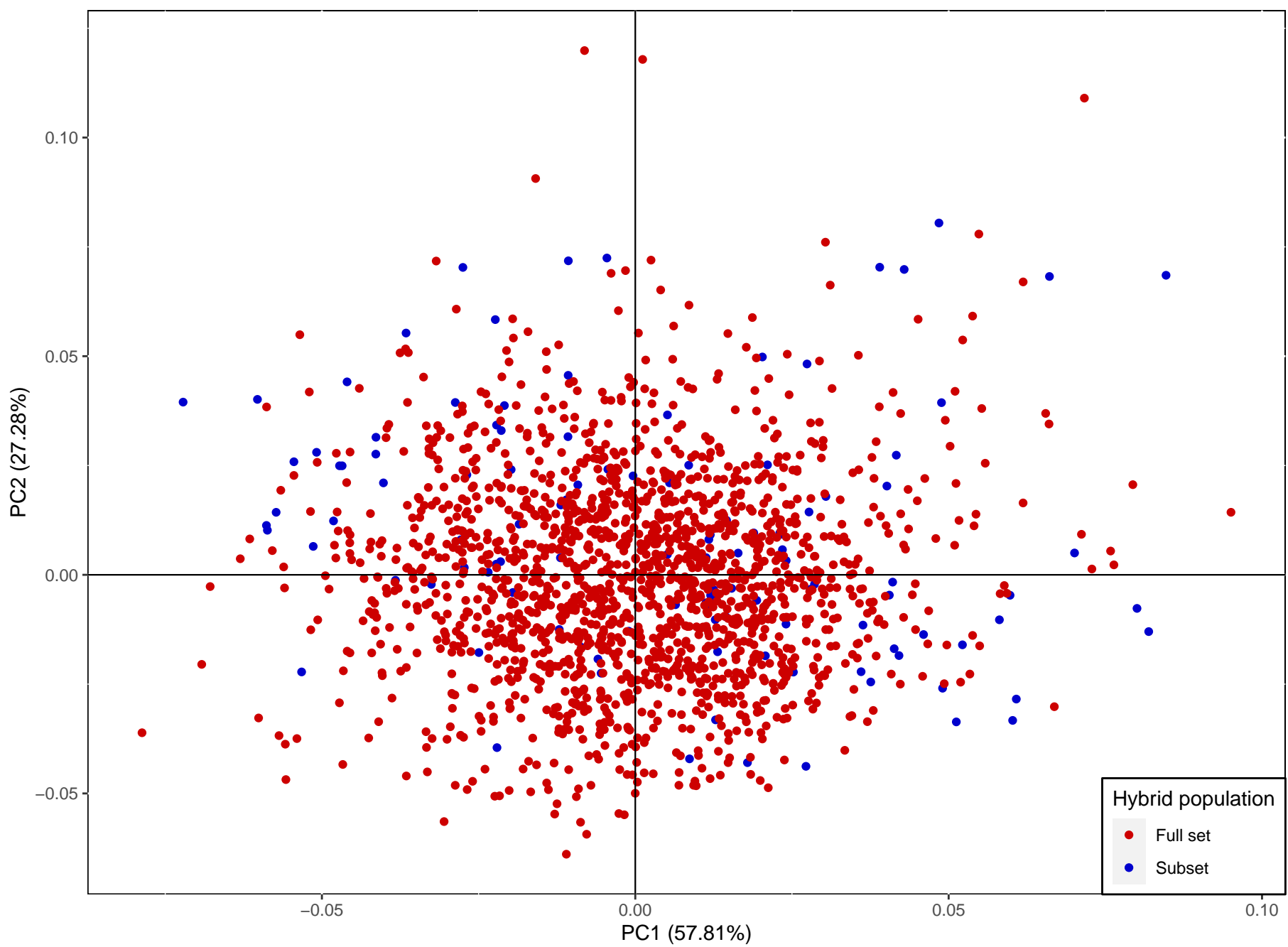

Supplement: Supplementary file 2 — Principal Component Analysis (PCA) of grain yield, protein content and sedimentation volume in the full set of 1.744 hybrids (red dots) initially tested in agronomic trials and the subset of the 119 selected hybrids thereof for in-depth quality analyses (blue dots). Supplementary file2 (PDF 17 kb) [file 122_2022_4039_MOESM2_ESM.pdf]
